# Soil Bacterial Diversity Screening Using Single 16S rRNA Gene V Regions Coupled with Multi-Million Read Generating Sequencing Technologies

**DOI:** 10.1371/journal.pone.0042671

**Published:** 2012-08-06

**Authors:** Sotirios Vasileiadis, Edoardo Puglisi, Maria Arena, Fabrizio Cappa, Pier S. Cocconcelli, Marco Trevisan

**Affiliations:** 1 Università Cattolica del Sacro Cuore, Faculty of Agricultural Sciences, Institute of Agricultural and Environmental Chemistry, Piacenza, Italy; 2 Institute of Microbiology, Piacenza, Italy; Catalan Institute for Water Research (ICRA), Spain

## Abstract

The novel multi-million read generating sequencing technologies are very promising for resolving the immense soil 16S rRNA gene bacterial diversity. Yet they have a limited maximum sequence length screening ability, restricting studies in screening DNA stretches of single 16S rRNA gene hypervariable (V) regions. The aim of the present study was to assess the effects of properties of four consecutive V regions (V3-6) on commonly applied analytical methodologies in bacterial ecology studies. Using an *in silico* approach, the performance of each V region was compared with the complete 16S rRNA gene stretch. We assessed related properties of the soil derived bacterial sequence collection of the Ribosomal Database Project (RDP) database and concomitantly performed simulations based on published datasets. Results indicate that overall the most prominent V region for soil bacterial diversity studies was V3, even though it was outperformed in some of the tests. Despite its high performance during most tests, V4 was less conserved along flanking sites, thus reducing its ability for bacterial diversity coverage. V5 performed well in the non-redundant RDP database based analysis. However V5 did not resemble the full-length 16S rRNA gene sequence results as well as V3 and V4 did when the natural sequence frequency and occurrence approximation was considered in the virtual experiment. Although, the highly conserved flanking sequence regions of V6 provide the ability to amplify partial 16S rRNA gene sequences from very diverse owners, it was demonstrated that V6 was the least informative compared to the rest examined V regions. Our results indicate that environment specific database exploration and theoretical assessment of the experimental approach are strongly suggested in 16S rRNA gene based bacterial diversity studies.

## Introduction

Use of the 16S rRNA gene as a bacterial evolution marker was a breakthrough for microbial ecology studies in the late 1980s [1]. Approaches like polymerase chain reaction (PCR) product screening of the 16S rRNA gene marker using environmental nucleic acid templates became common in soil microbial ecology [2]–[5]. Thus, shifting away research from strictly cultivation-based methods, and making possible to obtain information about bacterial community structures in their natural habitats.

The methodologies from the 90's, along with the new generation of high throughput screening of the 16S rRNA gene revealed that the bacterial diversity existing in just a few grams of soil was far more immense than previously believed [6], [7]. With the additional factor of the variability observed between soil environments, it became necessary to use multiple sample replicates and increased numbers of 16S rRNA gene amplicons (∼500,000 per gram soil) [6], [8]. Illumina sequencing, technology with abilities of generating multimillion partial 16S rRNA gene sequence reads is promising concerning meeting the throughput demands of soil microbial ecology studies at a reduced cost [9], [10]. However, contemporary technology limitations restrict the screened sequence length to stretches of a maximum of ∼230 bp, which is roughly equal to 16S rRNA gene singe hypervariable (V) regions.

The aim of the present study was to assess the use of Illumina sequencing for massive parallel screening of bacterial 16S rRNA gene diversity in soil environments based on the information potential of such short reads (single V region). 16S rRNA gene stretch for RDP database soil derived sequences was explored for conservation, and potential primer designing sites were proposed. Afterwards, four consecutive 16S rRNA gene hypervariable (V) regions were analyzed; namely V3, V4, V5 and V6. These sequences were examined by means of properties related to contemporary Illumina technology limitations. The performed tests included: (i) screening the suitability of V regions according to sequencing technology read length screening abilities; (ii) assessment of conservation of sequence stretches flanking the examined V regions; (iii) estimation of pairwise sequence distances as a means for evaluating how representative the trimmed V region is of the full-length 16S rRNA gene sequence; and (iv) taxonomy information loss of trimmed sequences as compared to their full length versions. Finally, a virtual experiment based on sequences and outcomes of previously performed studies was used to identify expected differences between V regions according to 16S rRNA gene sequence frequencies.

## Results

### Properies of soil derived 16S rRNA gene sequences

**Figure 1:**
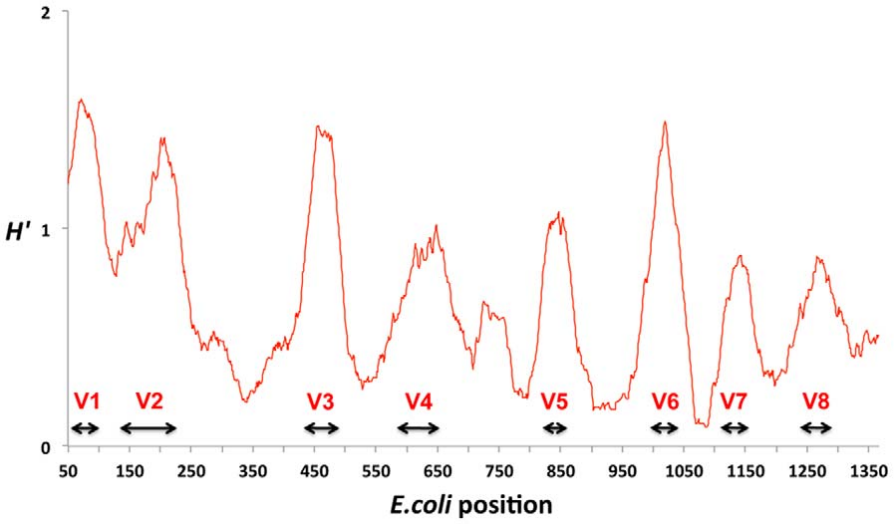
Entropy plot of 42,109 soil derived 16S rRNA gene sequence alignment. Hypervariable regions indicated as designated by Baker *et al.* [24] *E. coli* nucleotide numbering. Sequence area presented excludes poorly supported areas from the beginning and end of the sequences (due to nearly full sequences) and thus excludes the V9 region.

**Figure 2:**
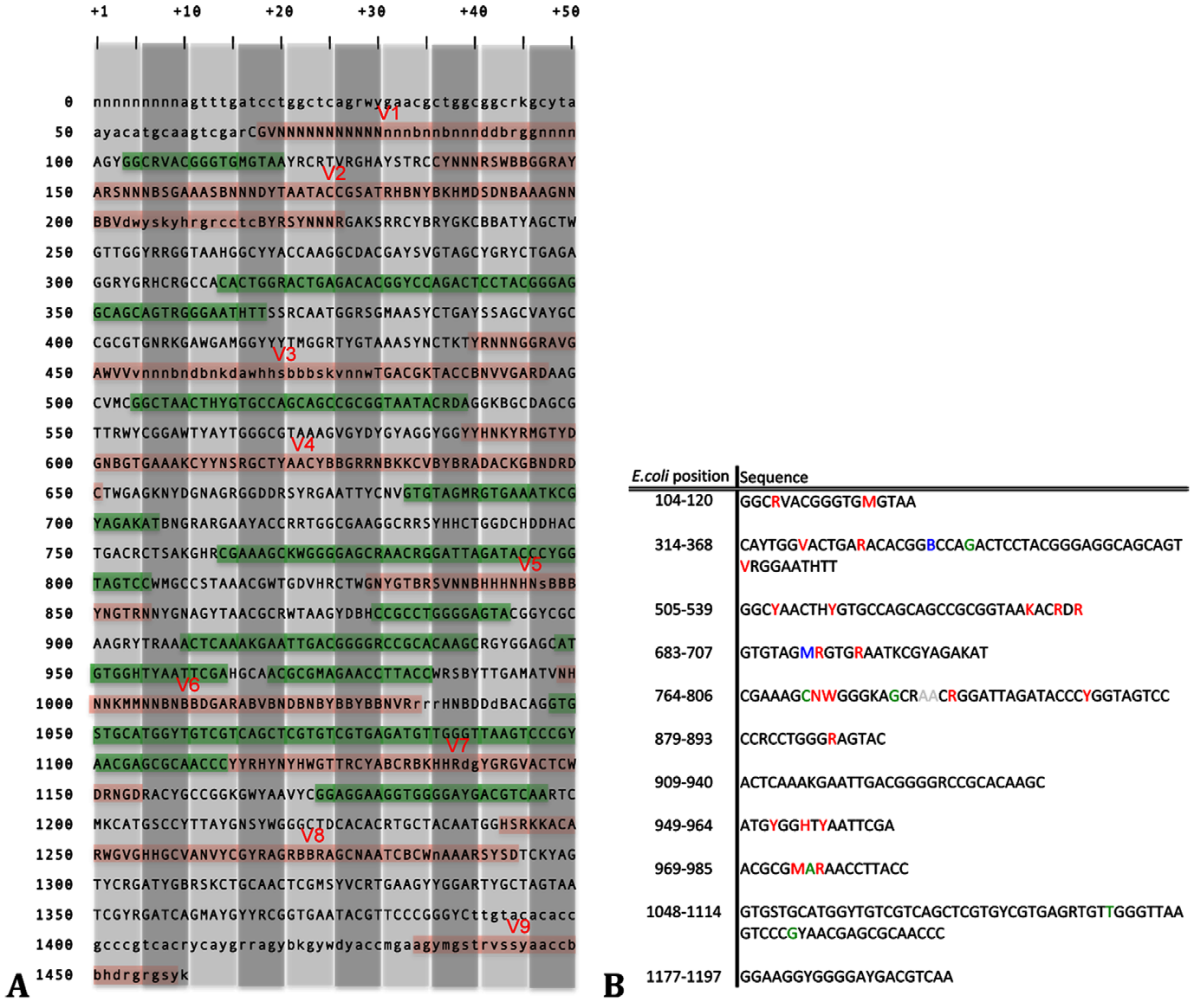
16S rRNA gene sequence conservation of soil derived sequences. A) Nucleic acid base composition of the 16S rRNA gene consensus sequence of the 41,109 RDP database soil derived sequences for 90% conservation cutoff value. Red background positions include hypervariable stretches as reported in reference [24] and expanded in the current study, while green background positions are proposed primer designing sites in reference [11]. The IUPAC system was used for denoting per base variability (degeneracies) and lower-case letters are used for nucleotide positions where gaps participated by more than 10% in the position throughout the sequence alignment. B) Comparison of present study results for 95% sequence conservation with the ones provided in reference [11] for 90% sequence conservation. Letter color coding referring to differences found on sequences of this study compared to that of reference [11]: red) increased variability; blue) altered degeneracy without variability increase; green) reduced variability; grey) although presence of two nucleotides in that position is implied in reference [11], these are missing in the published table.

**Figure 3:**
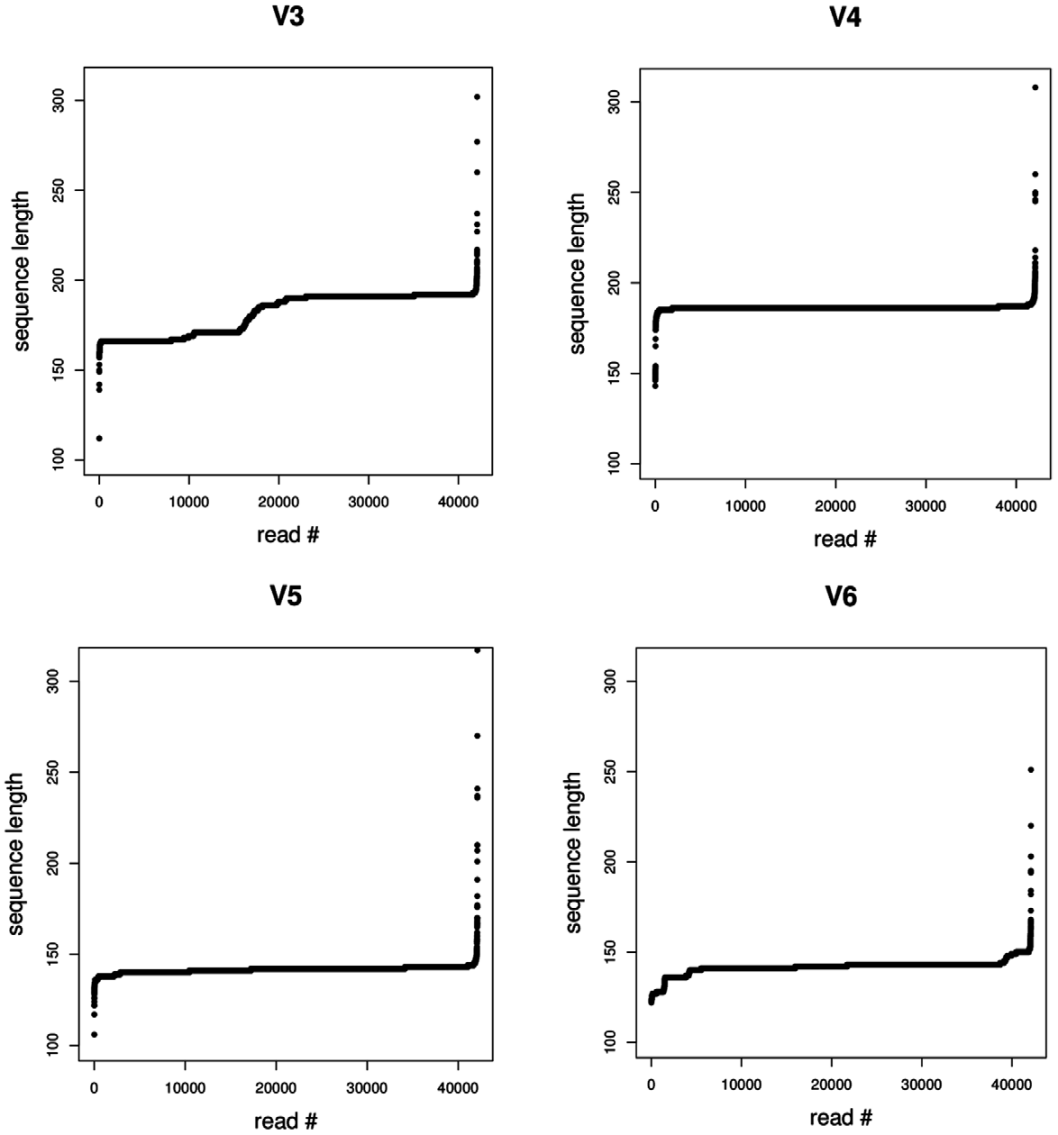
Distribution of commonly screened V region fragment lengths. Fragment lengths including the examined hypervariable regions for all screened (41,109) sequences. Sequence fragments were plotted according to length ascending order.

42,109 full or nearly full length 16S rRNA gene sequences derived from currently cultured and uncultured soil bacteria were used for performing the following analyses. Sequence conservation was examined using the Shannon entropy values (*H′*), while conserved sites flanking the hypervariable regions were also assessed concerning their suitability for designing primers. Out of the four selected V regions those showing the greatest variability were V3 and V6, and those with the longest V sequence lengths were V3 and V4 (Fig. 1 and 2). Stretches longer than 105 bp were identified as hypervariable for V3 and V4 while the corresponding value for V5 and V6 was slightly higher than 27–35 bp. Conservation screening of nucleic acid bases that were common for at least 95% of the examined sequences produced stretches with the potential for being selected as priming sites (green background color in Fig. 2). Identified potential amplicon lengths for the referred per primer coverage (or minimum 90% per primer-set) were: 175 bp (348–533 *E. coli* numbering) with maximum 3 degeneracies per primer for 18 bp primers or 190 bp (341–531 *E. coli* numbering) without degeneracies per primer for V3; 282 bp (516–798 *E. coli* numbering) with low primer degeneracies for V4; 108 bp (788–896 *E. coli* numbering) with low number of degeneracies per primer for V5; 137 bp (921–1068 *E. coli* numbering) with low number of per primer degeneracies for V6. When examined, regardless of the conservation of the various sites, and based on previously indicated sites [11], amplicon lengths were less than 200 bp for more than 99.8% of the amplicons for V3 and V4 and less than 150 bp for V5 and V6 (Fig. 3).

**Figure 4:**
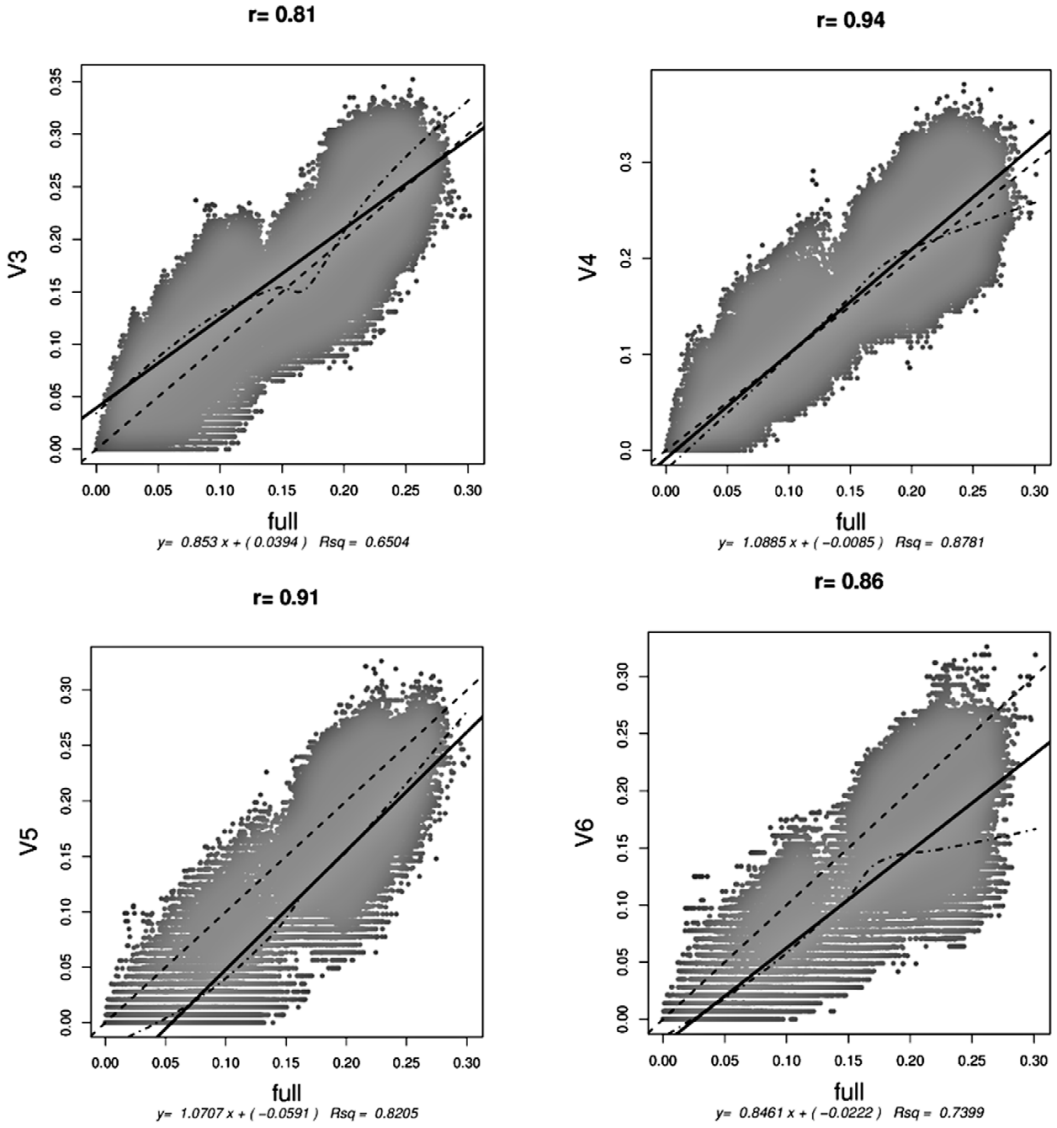
Pearson correlation tests between corresponding sequence distances of examined V regions and FL variants. All tests were significant (P<001). Test correlation index (r) values and linear models (presented with solid lines) used to describe overall trends are provided above and below each plot. Local relationships between corresponding sequence distances of the FL and other datasets are expressed with the non-parametric LOWESS (locally weighted regression and smoothing scatterplots) regression analysis plotting (dot-dashed lines), while the ideal y = x correlation is also plotted (dashed lines).

Effects of sequence length and V region variability patterns on obtained sequence distances were assessed by comparing distances of trimmed V region sequences with their full length variants (Fig. 4). Correlation tests showed V region datasets to perform in the following descending order: V4, V5, V6, V3. Overall trends were further assessed by linear model applications. Out of the four V regions, slopes closer to 1 were observed for V4 (R^2^ = 0.88) and V5 (R^2^ = 0.82). V3 and V6 slopes had values lower than one and applied linear models did not describe the data-points well. Linear model formulas indicate an over-estimation trend for V3 distances and a corresponding under-estimation for V5 and V6 for distances between 0 and 10%. The non-parametric locally weighted regression model analyses (LOWESS) showed consistency of the linear regression with local trends for sequence distances of the referred range. V4-FL comparison demonstrated an averaging distance consistency to up to sequence distances of 0.2.

**Figure 5:**
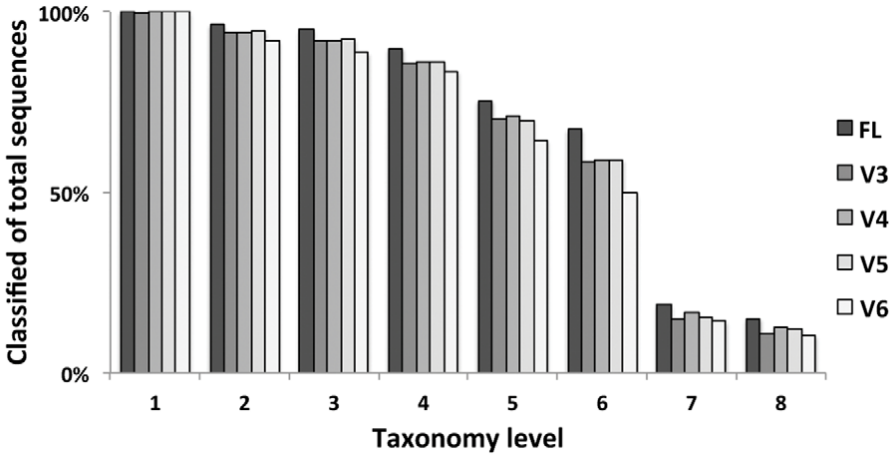
Taxonomy classification depth comparisons among V region datasets and the FL variants.

**Figure 6:**
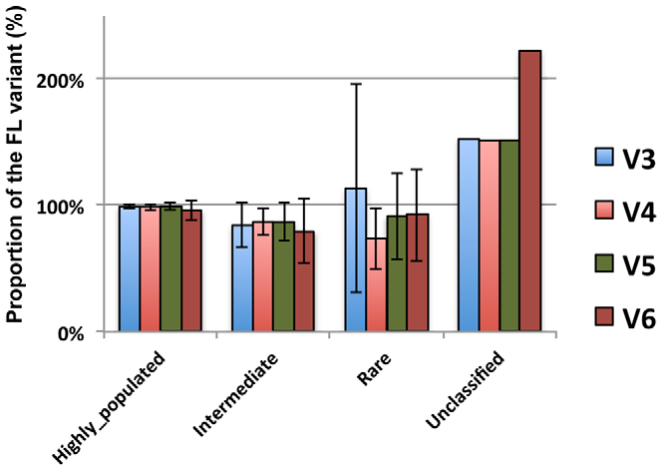
Over or under representation of phyla in the examined datasets. Values are expressed as percentage of the taxonomical annotations obtained for the FL sequence variants. Taxa were characterized into groups according to the existing sequence numbers as indicated in Table 1.

Classification depth testing indicated that all V region datasets showed a similar under-representation of existing sequences throughout all taxa per taxonomical level, with V6 performing worst of all (Fig. 5). Phylum level taxonomical classification differences between the full-length sequences and the V region trimmed variants were assessed considering obtained sequence numbers per phylum. Phyla were characterized as “highly populated”, “intermediate populated”, and “low populated” (or “rare”) according to the sequence numbers existing in each taxon as indicated in the footnote of Table 1 and the Materials and Methods section. Highly populated phyla of the dataset, were shown to be less affected by sequence trimming, than either the phyla encompassing 1000 or less sequences or the group containing the unclassified sequences (Table 1 and Fig. 6). Under-representation trends were observed for intermediate and low sequence numbers encompassing phyla, while over-representation by above 50% was observed for the unclassified sequences. V4 and V6 included classifications of highly populated phyla with a difference of greater than 5% in sequence content between the examined V region and the corresponding full-length variants. Main source of this reduced FL representation was the phylum of *Acidobacteria*. In intermediate populated phyla such differences existed for *Planctomycetes*, *Chloroflexi*, *Gemmatimonadetes* and *Nitrospira* that were under-represented for all V region datasets, while the TM7 was under-represented only for V3 and V5 and *Verrucomicrobia* along with *Cyanobacteria* were under-represented for V6. In rare phyla V3 and V5 had more bacterial phyla with differences smaller than 5% as compared to the full-length dataset, with *Chlamydiae* and *Fusobacteria* having smaller differences for all V region datasets. Analysis for faulty assignments of the highly and intermediate populated taxa showed that the overall effect of such events was low for all tested V regions (Figure S1). However, the unclassified group compositions demonstrated a reduced ability of all regions to contribute in identifying rare taxa and a lesser ability of V6 to identify all phylum “population” categories (particularly the highly populated *Acidobacteria*).

**Table 1:** Classification of the full-length sequences and their trimmed to the examined V region variants.

Group	Taxon	FL	V3	V4	V5	V6
**A**	**Proteobacteria**	15786	15875	15835	15678	15760
	**Firmicutes**	7388	7209	7431	7619	7144
	**Actinobacteria**	7373	7380	7292	7275	7353
	**Acidobacteria**	4607	4421	4350	4418	3781
	**Bacteroidetes**	1861	1870	1856	1836	1894
**B**	**unclassified**	1521	2318	2303	2307	3383
**C**	**Planctomycetes**	808	752	706	641	725
	**Verrucomicrobia**	759	759	730	749	688
	**Chloroflexi**	690	493	560	391	477
	**Gemmatimonadetes**	446	226	304	392	124
	**Cyanobacteria**	319	318	309	319	280
	**TM7**	162	146	154	153	169
	**Nitrospira**	114	96	95	106	101
**D**	**Deinococcus-Thermus**	97	91	89	98	58
	**OP10**	59	40	19	33	41
	**WS3**	45	36	19	22	9
	**Spirochaetes**	12	13	7	13	12
	**Deferribacteres**	11	9	9	6	8
	**BRC1**	9	11	8	7	7
	**OD1**	7	9	4	12	12
	**OP11**	7	3	3	7	6
	**Tenericutes**	7	7	6	6	6
	**Thermotogae**	5	5	5	7	6
	**Chlamydiae**	4	4	4	4	4
	**Chlorobi**	4	3	3	3	3
	**Synergistetes**	4	4	2	2	4
	**Aquificae**	2	8	2	2	3
	**Fusobacteria**	2	2	2	2	2
	**Lentisphaerae**	0	1	0	0	0
	**Thermodesulfobacteria**	0	0	0	0	2
	Taxa were categorized according to included sequence numbers or annotation to known taxa in RDP database and are denoted with different letters as shown here:					
	**Highly “populated” (>1000)**	**A**				
	**Unclassified**	**B**				
	**Intermediate “populated” (>100)**	**C**				
	**Low “populated” or “rare” (≤100)**	**D**				

### Simulated screening of soil samples using single V regions

Published soil bacterial 16S rRNA gene diversity datasets were downloaded and used as templates for generating corresponding virtual samples. The latter were used for assessing differences between V region trimmed fragments and their full length sequences, while used in taxonomy, OTU and phylotype screening approaches.

**Figure 7:**
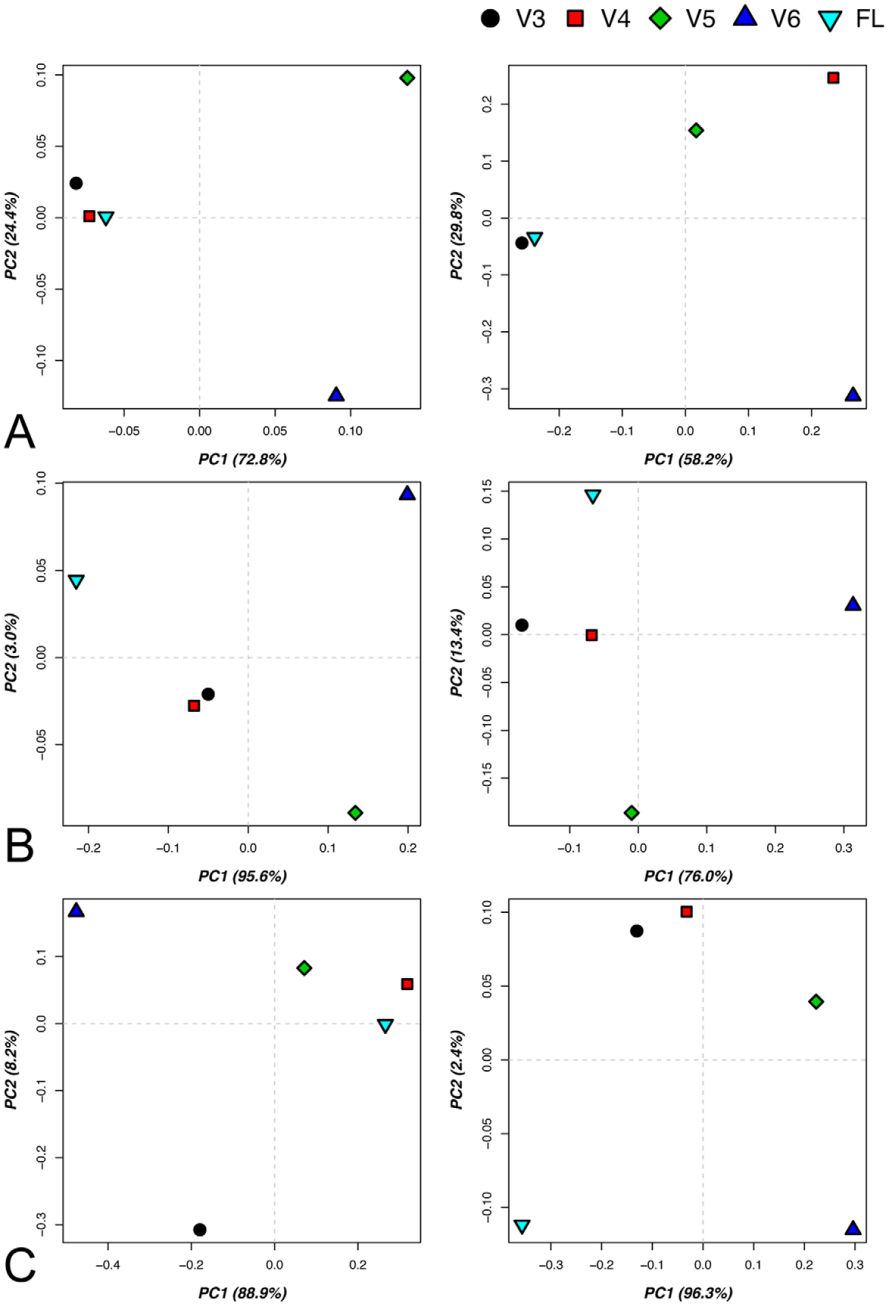
Taxonomy, OTU (3% sequence distance) analysis and Unifrac results of the performed simulation. A) PCA results of matrix generated by sample distances based on classified sequence relative abundance (left) and presence absence (right) for the V regions and FL datasets. B) Similarly to A for OTU relative abundance (left) and presence absence (right). C) PCA results for matrices generated using the weighted (left - phylotype relative abundance based) and unweighted (right - phylotype occurrence based) Unifrac analysis result distances between samples for the V regions and FL datasets.

Dataset topologies based on sample distances showed an overall better approximation of the FL dataset by the longer stretch V region datasets, V3 and V4 (Fig. 7). V3 showed the best clustering ability with the FL for both relative abundance and presence-absence taxonomical classification matrices, while V4 only coincided close to FL for the relative abundance matrices (Fig. 7 A). V3 and V4 also performed better than V5 and V6 in the OTU approach for both relative abundance and presence-absence matrices of OTUs (Fig. 7 B). Sample distances according to weighted and unweighted Unifrac results indicated that when relative abundance of reads is estimated V4 and V5 resided closer to the FL dataset (Fig. 7 C left). However, in the case that only sequence occurrence per sample was considered, sample distances for V4 and V3 more closely resembled the FL sample distances, but they did not reside as closely as in the previously mentioned approaches (Fig. 7 C right). Overall, V5 and V6 datasets had a poor performance with V5 being slightly closer to the FL than V6 according on the horizontal axes, where most of the variance is explained.

## Discussion

16S rRNA gene diversity screening using technologies like Illumina that produce multimillion sequence reads is a very appealing method for elucidating ecology concepts in complex environments such as soils. However, as indicated in the present study, there are several issues related to contemporary technology abilities and properties of screened environments that should be considered.

Sequence conservation is an important factor for determining the potential of screening depth of various taxa using an existing library. Our results (Fig. 2) differed from previous studies assessing conserved 16S rRNA gene areas that were based on representative sequences of the total RDP database. Although overall mapping of these areas on the selected reference 16S rRNA gene was consistent for most of the conserved screened nucleic acid bases, we identified a larger number of polymorphic sites than found before [11]. A potential explanation for this observation has to do with the fact that the RDP database deposited sequences are dominated by human microbiome related bacteria. A simple keyword search (e.g. “human” or “soil”) shows that in the RPD database about 56% of the ∼1,000,000 deposited 16S rRNA gene sequences that are longer than 1200 bp are derived from human body related environments. On the other hand, less than 5% of the database sequences are derived from soil. This is contradictory to the estimated number of species in the two environments. About 15,000 different species were identified in the complete human microbiome project, while more than 50,000 species were estimated to exist per gram of soil [6], [12]–[15]. Therefore, it is important to consider the particulars of the studied environment during experimental design, since it is connected to the diversity of existing niches.

Interconnected to the previous discussion point is the operational fragment length for an Illumina technology application. Current Illumina technology screening abilities according to the latest available (v4–v5) chemistries are maximized when using the Genome Analyzer IIx (GAIIx) and exploiting the paired-end reading ability (obtaining reads from both sequence fragment ends). It has been demonstrated that relatively good read quality results can be obtained for read-lengths of 125 nucleotides for each of two reads per fragment (with the second read showing lower qualities at the error prone read ends) [9]. Assembly of the paired-end reads per sequenced amplicon in previously published studies required a minimum of 5–12 nucleotides of read overlap [9], [10], [16], which reduces the operational amplicon length to a maximum of 226 bp. Moreover, our attempt to screen RDP sequences for potential tandem repeats that might interfere with assembly at the overlapping regions did not indicate that related problems would exist by selecting the option of a 10 nucleotide overlap (data not shown). Therefore, the 226 bp of amplicon screening seems like an upper limit concerning length influence on screening abilities, yet multiplexing is the major objective of technological applications and this requires the addition of barcode sequences in at least one of the two primers used. Proposed multiplexing methods involve: a) primer indexing by addition of a few unique bases on the 5′ end of one (or both) of the amplification primers plus a 2 bp linker sequence for reducing potential index stretch effects on reaction specificity during environmental sample PCR performance [10], [16]; b) use of primers with 5′ extensions with Illumina sequencing adapters, plus an index sequence [9] that enables a third sequence read (in paired-end reads usage) for identification of barcodes and does not affect the operative sequence read length (similar philosophy to that of Illumina multiplexing kits [17]). All these methods have their advantages and disadvantages but all of the approaches result in restriction of amplicon screening abilities to maxima of approximately 215 bp of length. This screening length was indicated as being sufficient for screening all V regions with less than 0.5% information loss. However, 16S rRNA gene conservation around V4 indicated that robust primer designing for such short amplicons (based on reference [11]) is difficult to achieve for soil environmental samples.

RDP database soil derived sequences were further analyzed for assessing representation of the tested full-length sequences concerning obtained distances and taxonomy annotations during sequence comparisons, when sequence parts belonging in the tested V-regions are used. Correlation tests of generated distances of sequences belonging to the same strains for the full length sequences and their V region variants, showed an overall superior performance for the V4 region dataset, followed by V5 for both the Pearson correlation values and the dispersal of points around the applied linear model. However, when examining more carefully V region datasets, for distances of 0–13% according to FL dataset distances there appears to be a distance overestimation for V3 and an underestimation for V5 and V6. This indicates that more per base variability is accumulated in the V3 region than in the other V regions and the corresponding section in the FL sequence. Higher resolution of signature sequences can therefore be obtained at the referred OTU definitions.

Taxonomy classification of the V region and FL datasets indicated that there is some information loss along with sequence size reduction, particularly for the V6 dataset (Fig. 5). However, sequence classification was equal or above 70% of the total reads and above 90% of the FL classified sequences for the V3, V4 and V5 datasets even in the case of taxonomical level 5 (encompassing order, suborder and family level classifications). Thus, use of these regions provides may facilitate relatively thorough screening of taxa related to large part of the global biogeochemistry of natural environments. According to phylum level analysis results, observed taxonomical information loss of V region datasets (thus resulting in the increase of the unclassified group of sequences) was mainly derived from intermediate populated or rare phyla of the reference database. In this analysis the V6 dataset had more than twice the FL dataset unclassified sequences, while the other V region datasets had approximately 1.5 times the unclassified FL sequences. The fact that less populated phyla were also under-represented during classification is partly due to the reference database composition. Low representation of taxa in the reference database affects the classification confidence and the probability of identification via partial sequence read (word) matches while searching for closest sequences with the naïve Bayesian classifier [18].

The performance of the simulated analysis provided an approximation of the effect that sequence relative abundance and richness in environmental soil samples would have on diversity assessment. Overall it was shown that datasets of V-regions encompassing longer sequence stretches (V3 and V4) generated sample distances more similar to the ones produced by the FL dataset compared to V5 and V6. Such differences between the V3, V4 and the V5 dataset were not indicated in the database screening analyses performed in the first part of this study. That is possibly because of the composition of the tested soil microbiomes, having increased relative abundance of sequences showing performance differences when the trimmed V5 or V6 regions are compared with their full-length sequence variants.

### Concluding remarks

Combination of Illumina sequencing technology with screening partial 16S rRNA gene sequence reads in environmental samples can be a powerful tool for microbial ecology studies. However, this combination has some limitations as a result of the sequence screening length. V3 region selection as the screened 16S rRNA gene stretch did not perform as well as when the non redundant soil derived sequence dataset was screened, but it had a superior performance when sequence frequencies came close to those found in soil environments. V4 had a high overall performance, but compared to the rest it had a reduced conservation of flanking sequence sites of the V region. This lack of conservation may be restricting concerning diversity screening depths. V5 had a desireable diversity screening depth and an overall good performance for the non redundant dataset, but the information extracted from this region showed differences with the full-length 16S rRNA gene sequence variants in the non-redundant dataset. Thus showing the effect of the composition of the tested bacterial communities to the outcome of the V5 selection approach. V6 was outperformed in all tests apart from the one of flanking sequence conservation.

Collectively, these results suggest that partial 16S rRNA gene sequence reads corresponding to single V regions have flaws compared to their FL variants in soil bacterial community studies. Nevertheless, some appear to capture the FL sequence information in a great degree. V3 properties can match the demands of many of total soil bacterial community screening studies. V5 on the other hand, is a relatively well performing representative of the shorter V regions. The shorter V regions can provide the opportunity of assessment of the sequencing quality of the reads used, since longer read parts of the sequenced amplicon strands overlap during assembly (and therefore agreement of base calling quality of the overlapped parts is examined), which is performed as part of the reconstruction of the screened V region sequence.

Incorporation of database exploration during initial experimental setup stages is strongly suggested for strategy improvement towards experimental goals. This especially holds true during primer designing phase, which is crucial concerning the quality of the produced data. Careful selection of template sequences for the primer designing process can improve primer-set collections for highly diverse environments like soil. Potentials for further methodology improvements and can be found in approaches like the use of more than a single V region screening or even the usage of multiple housekeeping genes [19]. However, it must be acknowledged that part of the power of the combination of bacterial 16S rRNA gene screening with Illumina sequencing is relying on the extensive existing full or nearly full gene length related databases, something that is lacking to some degree for other genes.

## Methods

**Figure 8:**
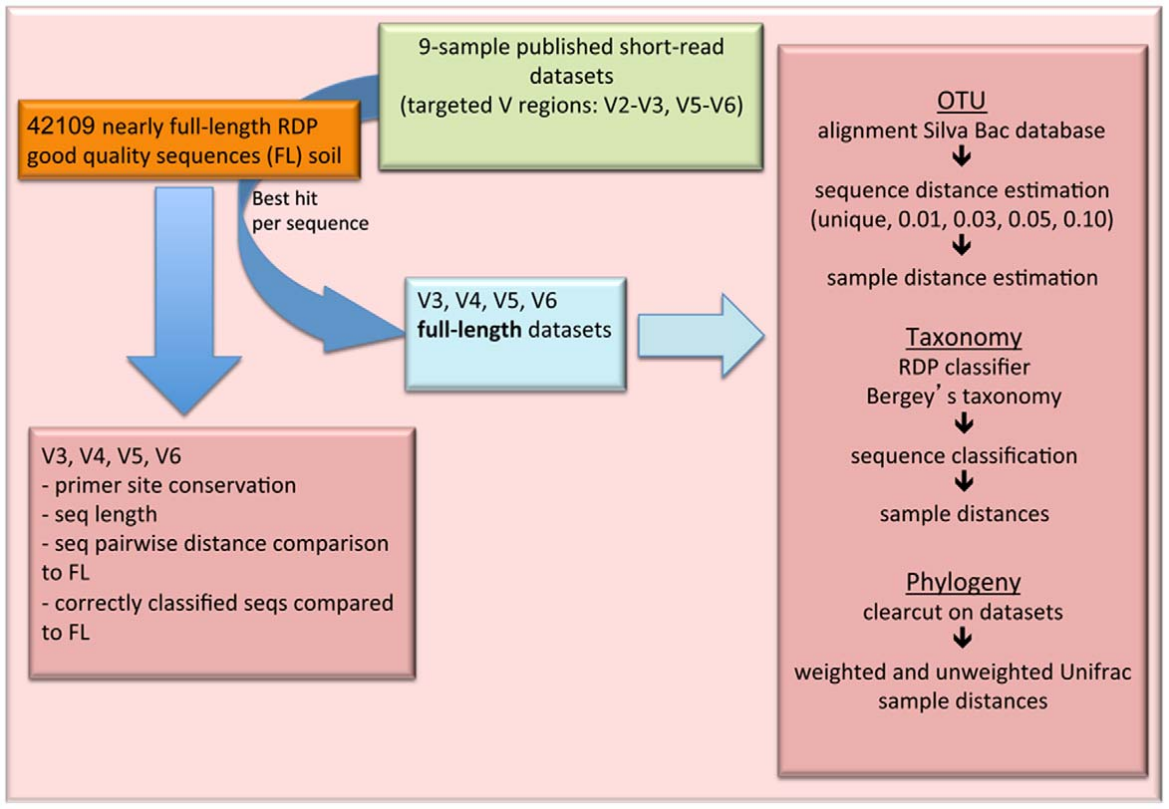
Study methods overview.

An overview of the approach is provided in Fig. 8.

### V region properties of soil derived 16S rRNA gene sequences

Datasets description: 42109 full or nearly full-length (≥1200 bp) soil-derived, ribosomal database project (RDP) database [20] 16S rRNA gene sequences of cultured and uncultured bacteria comprised the core dataset used in the comparisons of the hypervariable regions with the complete stretch of sequence reads. The *Escherichia coli* type strain (ATCC 11775T) 16S rRNA gene sequence with Genebank accession number X80725 was added in the datasets and was used as reference during analysis. Sequences were aligned using the NAST algorithm [21] with the amendments and the default aligner parameters reported in reference [22], while the pre-aligned SILVA bacterial 16S rRNA gene sequence set [23] was used as reference alignment. For consistency with the widely accepted *E. coli* 16S rRNA gene nucleotide position numbering [11], [24], this sequence was aligned with the *E. coli* 16S rRNA gene sequence used in these studies (Genebank accession 1VS5_A) and nine gaps were introduced in the sequence beginning in all cases where numbering of positions is referred. Aligned sequences were trimmed according to reference sequence positions 338–534 (V3), 515–700 (V4), 786–926 (V5), 1052–1193 (V6) for generating the desired hypervariable region datasets. Trimming positions were based on previously reported high coverage primer sites [11], while the final trimmed sequences encompassed these flanking primer designing sites.

Analysis of 16S rRNA gene conservation and V region lengths: Assessment of alignment based soil bacterial 16S rRNA gene sequences positional variability, was carried out by estimating the Shannon entropy (*H*′) values per nucleic acid base position. Gap positions existing in the reference sequence were removed from all aligned sequences and the *H′* values were calculated. An entropy plot was generated with plotted values per *E. coli* reference sequence position along the x axis, which were the result of the average *H′* value of 20 consecutive base positions. Moreover, the consensus sequence of all soil 16S rRNA gene sequences was generated using a 90% conservation cutoff value (degeneracies are indicated according to the IUPAC annotation system), and a 95% cutoff for identifying highly conservative priming sites. Results of conserved sites were compared to the previous study of Wang and Qian [11]. *H′* calculations were carried out with the bio3d package [25] executed in R software [26], while the nearly full 16S rRNA gene consensus sequence was calculated using the Mothur software [27]. Finally, examined hypervariable regions were also tested for stretch length distribution across all soil-derived RDP database sequences, for assessing their potential usage according to Illumina limitations.

Corresponding sequence distances and taxonomy comparisons between V region and FL datasets: Properties related to two major microbial diversity assessment approaches (OTU and taxonomy based) were examined in comparison to the respective properties of the full-length sequence variants. OTU and taxonomy based analyses were carried out using the Mothur software. Using the average linkage algorithm, distances between aligned sequences having the same identifiers were calculated and concomitantly compared for all V region datasets against the full-length sequences. Due to computational power limitations a subset of ∼10,000 sequences per dataset (ones derived from agricultural and grassland soils) was used generating ∼100,000,000 pairwise distances. Comparisons for 1,000,000 randomly selected distances per dataset corresponding to the same strain of origin, were used for performing Pearson correlation tests between each V region dataset and the full-read length variant. Taxonomy information differences throughout all datasets and the full-length sequence annotations were assessed using the naïve Bayesian classifier for 50% confidence resulting from bootstrap analysis [28], according to RDP taxonomy annotations that are consistent with Bergey's manual standards, using the SILVA database as reference. Sequence classification depth for all taxonomical levels and also over- or under-representations at phylum level for the hypervariable region datasets compared to the full-length sequences were reported. For the latter, taxa were divided into four categories according to the population of the database in sequence numbers, also taking into account whether or not sequences were classified. These categories were “highly populated” (>1000 sequences for participating taxa), “averagely populated” (above 100 sequences and up to 1000 per participating taxon), “scarcely populated” (less than 100 sequences) and “unclassified”. ANOVA was performed for estimating the significance of differences between the referred groups. The unclassified sequences group and cases of phyla with miss-identified sequences in the V regions datasets not existing in the original full-length dataset were excluded from the test. The Shapiro normality test and the Levene's test of equality of variance were performed for assessing if ANOVA conditions were met. The Shapiro normality test showed that this condition was not met for all examined groups and the non-parametric Nemenyi-Damico-Wolfe-Dunn joint ranking test (for confidence intervals of 99%) with Tukey test for pair-wise comparisons was applied using the Coin package [29] of R software.

### Environmental sample analysis simulation

Datasets description: Nine datasets in total, derived from soil bacterial 16S rRNA gene diversity screening results of previous studies using pyrosequencing, were used as templates for these analysis series. Major criteria for their selection were the range of sequence numbers per sample (26,000 to 54,000) and the read qualities. Studies with a corresponding dataset or sequence accession numbers used were: Roesch *et al.* [7] sequence accessions EF222481-EF248596, EF248597–276844, EF308591–362836; Will *et al.* [30] archive accessions SRR059809, SRR061002, SRR061009; Nacke *et al.* [31] archive accessions SRR064358, SRR064370, SRR064374. Best matches of sequences derived from these files in the SILVA bacterial 16S rRNA gene reference alignment sequences [23] according to the NAST algorithm performance [21], [22] were extracted and comprised the full read length replacements for each sequence in the nine-sample dataset used for concomitant analyses (referred as test dataset). The aligned sequence test dataset version was trimmed to the examined V regions (as referred to in V region analysis). Gap removal of the full length or trimmed sequences lead to the generation of 5 datasets containing: the full-length (FL), V3, V4, V5 and V6 variants of the test sequence dataset.

Data analysis: V region performance was assessed by means of Classification, operational taxonomic unit (OTU) and phylogenetic results for each of the V region dataset comparisons with the FL dataset. For the classification based analysis (performed with the parameters described above) sequences of each dataset were classified and sample distances were calculated using the Bray-Curtis transformation for relative abundance matrices and the Jaccard transformation for presence absence matrices. The obtained pairwise distances were used as loadings for performing PCA analysis in corresponding sample distances between generated datasets. Using the same methods, sample distances generated by an OTU approach for OTU definition of 3% sequence distances were used for OTU assessment differences between generated datasets (V-regions and FL) for relative abundance and presence absence matrices. The phylogeny based analysis included calculations of dataset distances based on obtained sample distances per dataset as calculated by encompassed sequence evolutionary relationships. The initial step was to perform a relaxed neighbor joining algorithm performed by the Clearcut application [32] for producing the phylogenetic tree that was concomitantly used for calculating sample distances using weighted and unweighted Unifrac analyses [33], [34]. Sample distances were used for generating one matrix for weighted distances and one for un-weighted distances for all the datasets and matrices that were analyzed with PCA analysis.

## Supporting Information

Figure S1 **Analysis of annotations for high and intermediate “populated” taxa, and unclassified sequences as defined in the footnote of** **Table 1** **.** Bars presented for each dataset correspond to the relative participation of obtained annotations, while each bar is colored according to the relative proportions of original FL annotations.
(TIF)
