# Correction: Soil Bacterial Diversity Screening Using Single 16S rRNA Gene V Regions Coupled with Multi-Million Read Generating Sequencing Technologies

**DOI:** 10.1371/annotation/bf36b938-523b-4c9f-ae86-16db7e8d39c1

**Published:** 2012-08-10

**Authors:** Sotirios Vasileiadis, Edoardo Puglisi, Maria Arena, Fabrizio Cappa, Pier S. Cocconcelli, Marco Trevisan

There is information missing from the Funding statement. The complete funding statement is: This study was financially supported by the SNAC project (project number 2011-1088, funded by Cariplo Foundation, Italy), the GENOBACT project (grant number G41J10000400002, sponsored by Lombardia Region, Italy) and was also supported by the Doctoral School on the Agro-Food System (Agrisystem) of the Università Cattolica del Sacro Cuore (Italy). The funders had no role in study design, data collection and analysis, decision to publish, or preparation of the manuscript. 

