# Supplementary figures and images for: Soil Bacterial Diversity Screening Using Single 16S rRNA Gene V Regions Coupled with Multi-Million Read Generating Sequencing Technologies
Source: PLoS One. 2012 Aug 6;7(8):e42671. doi: 10.1371/journal.pone.0042671 (PMC3412817; doi:10.1371/journal.pone.0042671)

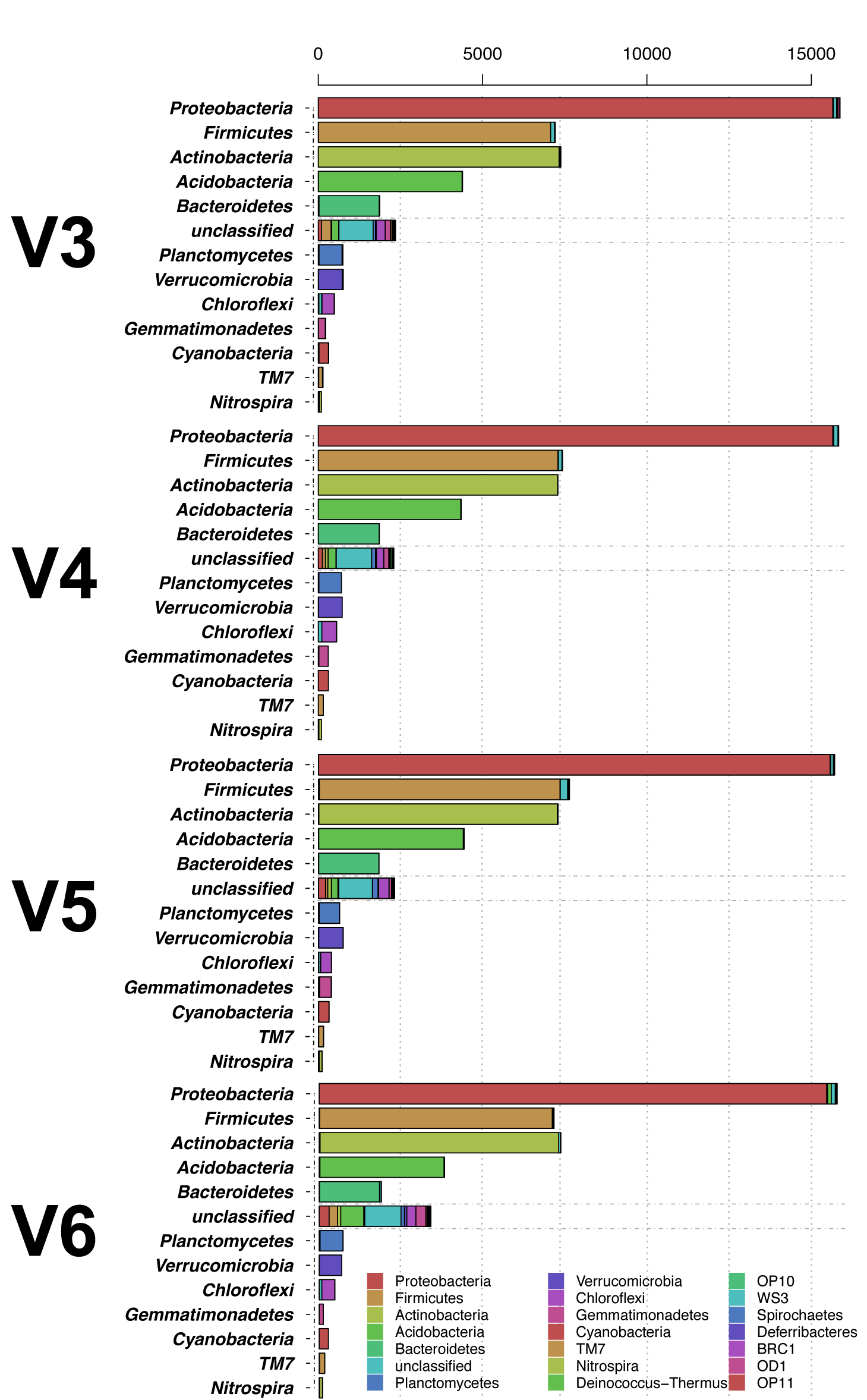

Supplement: Figure S1 — Analysis of annotations for high and intermediate “populated” taxa, and unclassified sequences as defined in the footnote of Table 1 . Bars presented for each dataset correspond to the relative participation of obtained annotations, while each bar is colored according to the relative proportions of original FL annotations. (TIF) [file pone.0042671.s001.tif]
